# Redo-Transcatheter Aortic Valve Replacement: Current Evidence and Procedural Considerations

**DOI:** 10.3390/jcm14186608

**Published:** 2025-09-19

**Authors:** Raviteja R. Guddeti, Hanad Bashir, Puvi Seshiah, Nadia El-Hangouche, Dean J. Kereiakes, Santiago Garcia

**Affiliations:** Interventional and Structural Cardiology, The Carl and Edyth Lindner Research Center, The Christ Hospital, Cincinnati, OH 45219, USA; hanad.bashir@thechristhospital.com (H.B.);

**Keywords:** aortic stenosis, aortic regurgitation, bioprosthetic valve dysfunction, cardiac computed tomography angiography, redo-transcatheter aortic valve replacement

## Abstract

Transcatheter aortic valve replacement (TAVR) has revolutionized the management of severe aortic stenosis (AS), with indications expanding from high and intermediate- to low-surgical risk patients. However, the durability of transcatheter heart valves (THV) is still an ongoing concern, especially in younger, low-risk patients. Redo-TAVR (TAVR-in-TAVR) is feasible and is associated with favorable short-term outcomes in observational studies and registries. Understanding native aortic valve (AV) anatomy and the characteristics of the index THV is critical to the technical and procedural success of redo-TAVR. Cardiac computed tomography angiography (CTA) plays a central role in pre-procedure planning and aids in appropriate valve selection and procedural planning in addition to identifying patients at high risk for coronary occlusion during redo-TAVR. While evidence on redo-TAVR is limited to retrospective observational studies, prospective registries will shed light on the long-term clinical and hemodynamic outcomes.

## 1. Introduction

Transcatheter aortic valve replacement (TAVR) has fundamentally changed the landscape of severe aortic stenosis (AS) management, emerging as a less invasive alternative to surgical aortic valve replacement (SAVR). Since the first case of TAVR by Cribier et al. in 2002 [1], there has been a paradigm shift in the evolution of TAVR. Refinements in patient selection, operator training, and preprocedural planning by utilizing advanced imaging as well as transcatheter valve miniaturization have contributed to improved procedural safety and long-term outcomes of TAVR. Robust clinical trial evidence has been pivotal in driving the expansion of TAVR indications [2,3,4,5]. Major international guidelines have endorsed TAVR as a Class I indication for symptomatic severe AS in patients at high or intermediate surgical risk. The most transformative shift occurred in 2018 with the presentation of the PARTNER 3 and Evolut Low-Risk trials, which evaluated TAVR in patients at low surgical risk [6,7]. These landmark studies demonstrated non-inferiority and potential superiority of TAVR compared to SAVR in key clinical outcomes, including early mortality, stroke, and rehospitalization. As a result, TAVR became a viable and often preferred alternative to surgery, even among younger and low-risk patients who were traditionally managed with SAVR. Most recently, the balloon-expandable SAPIEN 3 transcatheter heart valve (THV) (Edwards Lifesciences, Irvine, CA, USA) has been approved for treatment of asymptomatic severe AS, further expanding the use of this technology. All bioprosthetic valves, whether surgical or transcatheter, inevitably undergo structural degeneration. As TAVR indications expand to younger and lower-risk AS populations [6,7], consideration should be given to ‘lifetime management’, as most of these patients may outlive the durability of transcatheter heart valves (THVs). While evidence regarding the long-term durability of THVs is limited, redo-TAVR (TAVR-in-TAVR) or TAVR in bioprosthetic SAVR, along with explantation of THV, will need to be considered. It should be acknowledged that the risk profile of the patients may have changed considerably since the index TAVR procedure, making SAVR with THV explantation a less attractive option. In this review, we explore pre-procedural, procedural, and post-procedural considerations for redo-TAVR and current evidence.

## 2. Pre-Procedural Aspects of Redo-TAVR

### 2.1. THV Durability

As TAVR extends into younger and lower-risk populations, prosthetic valve durability has emerged as a critical concern. Unlike earlier cohorts, often elderly and with limited life expectancy, today’s TAVR recipients are increasingly expected to outlive their initial bioprosthesis.

### 2.2. Bioprosthetic Valve Dysfunction (BVD)

BVD encompasses several mechanisms, including structural valve deterioration (SVD), nonstructural valve deterioration (NSVD), leaflet thrombosis, and prosthetic valve endocarditis—each with distinct pathophysiology and clinical implications [8,9,10]. SVD is the most common and involves intrinsic leaflet damage, while NSVD includes paravalvular leak and valve malposition [8,9,11,12,13,14]. Leaflet thrombosis, often subclinical, may impair valve function but is often reversible with anticoagulation [15,16]. Though less frequent, prosthetic valve endocarditis remains a serious complication with high morbidity.

Valve durability is most often affected by SVD, which encompasses progressive leaflet degeneration, including calcification, fibrosis, thickening, and impaired leaflet mobility, leading to hemodynamic compromise. Mid-term studies have shown reassuring outcomes, with clinically significant SVD reported in only 5–10% of patients at 5 to 8 years post-implantation [17,18]. However, longer-term data beyond 10 years are lacking. Procedural variables can also compromise durability. Under expansion due to heavy annular calcification, asymmetric or canted deployment, and inaccurate sizing may lead to early degeneration or suboptimal leaflet function. Moreover, subclinical leaflet thrombosis, characterized by hypoattenuated leaflet thickening (HALT) and reduced motion on imaging, may impair durability despite often being asymptomatic.

A multifaceted approach, involving refinement in valve technology, optimized implantation technique to maximize valve expansion, careful patient selection, and individualized antithrombotic therapy, is essential to minimize BVD and ensure optimal valve durability [8,19].

## 3. TAVR Explantation

Management of SVD involving THVs includes either surgical explantation of THV followed by SAVR or redo-TAVR. The choice of strategy depends on patient demographics, anatomy, and underlying comorbidities. THV explantation is associated with increased short-term risk, including mortality. In the EXPLANT-TAVR International registry, 30-day and 1-year mortality rates were high at 13.1% and 28.5%, respectively [20]. Stroke rates were also high at 18.7% at 1 year. Urgent or emergent TAVR explant was performed in >50% of patients. Interestingly, in 34% of the patients, redo-TAVR was not feasible due to unfavorable anatomy and/or suboptimal results with redo-TAVR requiring urgent surgery. In the EXPLANTORREDO-TAVR global registry, TAVR-explant was associated with higher 30-day (13.6% vs. 3.4%; *p* < 0.001) and 1-year (32.4% vs. 15.4%; *p* = 0.001) mortality compared to redo-TAVR [21]. On landmark analysis, mortality was similar between the groups after 30 days. In addition, there was no significant difference in outcomes between patients who underwent the 2nd TAVR with either a balloon expandable (BEV) or self-expanding valve (SEV). However, most low-risk patients who underwent index TAVR at relatively younger ages may be considered high risk for surgical options for BVD due to advanced age and increased risk profile. While redo-TAVR may be feasible, pre-procedural imaging may help determine which patients are candidates for THV explantation.

## 4. Role of Cardiac Computed Tomography Angiography (CTA) Imaging in Redo-TAVR Planning

Similar to TAVR-in-SAVR, cardiac CTA plays a crucial role in redo-TAVR planning. Understanding native anatomy and the index THV device characteristics, including the type and size of the prosthesis, and the mode of THV failure, is central to the procedural success of redo-TAVR. The location and extent (nadir and the top) of the prosthesis leaflets in relation to the stent frame of the index THV are available for all the prosthesis [22]. These characteristics determine the potential depth of implantation of the 2nd THV device, depending on the mode of failure. Notably, for a prosthesis with stenosis, the second THV should ideally cover as much of the native leaflet as possible to avoid or mitigate leaflet overhang. On the other hand, for a regurgitant THV, although it is important to optimize index valve leaflet entrapment, one can afford a deeper deployment as it will address the mechanism of failure. This is especially true for THVs with a supra-annular design.

THVs are categorized into either SEV or BEV. However, for redo-TAVR planning, it is also recommended that valve types be categorized into short and tall frames. Consequently, TAVR-in-TAVR combinations include short-in-short, short-in-tall, tall-in-tall and tall-in-short combinations.

**Figure 1:**
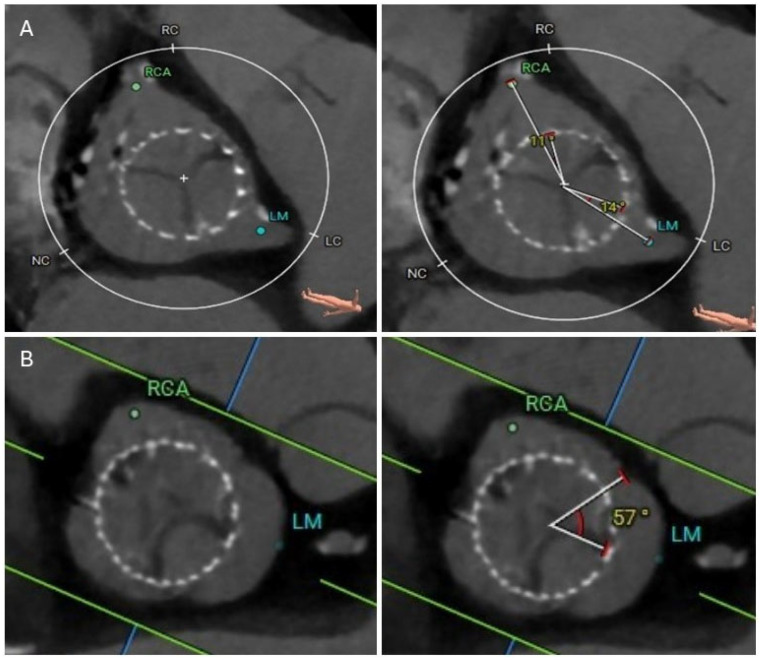
Cardiac CTA showing optimal commissural alignment (0–15 degrees) in a patient with 26 mm Evolut Pro index THV (**A**) and severe misalignment (45–60 degrees) in a patient with 29 mm Evolut Pro THV (**B**). Severe misalignment of the index THV can potentially result in difficult coronary access after redo-TAVR. CTA, computed tomography angiography; LC, Left coronary cusp; LM, Left main; NC, Noncoronary cusp; RC, Right coronary cusp; RCA, Right coronary artery; TAVR, transcatheter aortic valve replacement; THV, transcatheter heart valve.

**Figure 2:**
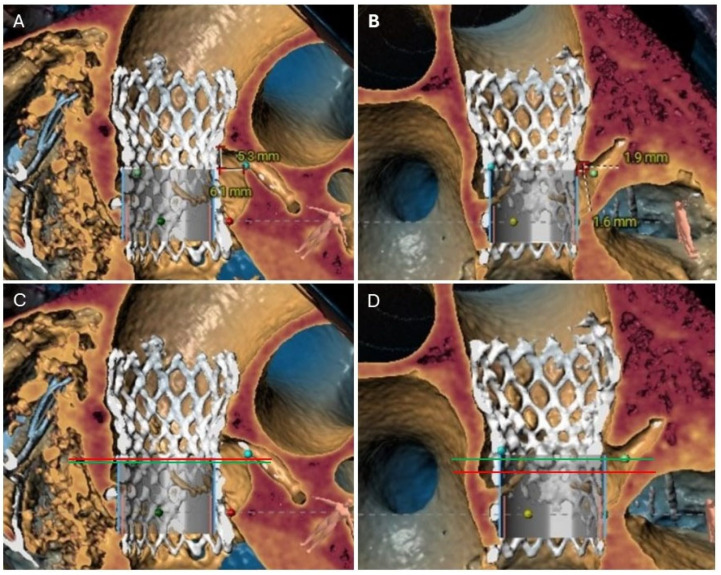
(**A**) 3D cardiac CTA demonstrating adequate VTC distance from the virtual S3 valve to LM. (**B**) On the contrary, the VTC distance to RCA is <2 mm, indicating a high risk for coronary occlusion if S3 is implanted at node 5 or above. (**C**) For LM, the NSP (green line) is at the level of CRP (red line), suggesting low risk for coronary occlusion. (**D**) However, for RCA, the NSP (green line) is above the CRP (red line), and coronary protection is recommended during redo-TAVR, especially with a low VTC on the right. CTA, computed tomography angiography; CRP, coronary risk plane; NSP, Neoskirt plane; LM, left main; RCA, right coronary artery; VTC, valve to coronary.

**Figure 3:**
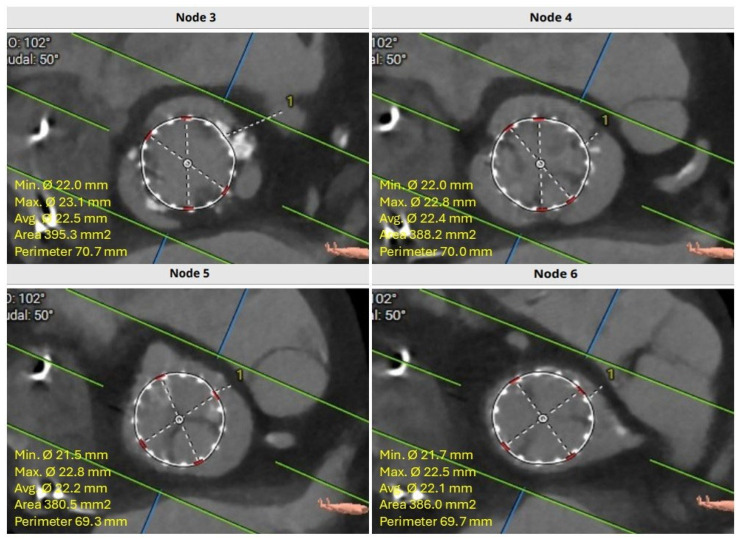
Cardiac CTA demonstrating index THV stent frame dimensions at multiple levels. CTA, computed tomography angiography; THV, transcatheter heart valve.

After a careful review of the native anatomy and the index THV properties, the first step would be to determine coronary alignment (Figure 1). The risk of coronary compromise is higher with coronary misalignment, and the potential for leaflet modification is also limited in that situation. It is also important to rule out leaflet thrombosis as the etiology of the prosthesis degeneration, as the treatment may differ (i.e., anticoagulation). The second step would be to measure the in vivo stent dimensions at various levels depending on the index prosthesis type. Valve-to-aorta (VTA) distance should be measured at various levels: valve-to-coronary (VTC), valve-to-Sinus of Valsalva, and valve-to-sinotubular junction (STJ; VTSTJ) (Figure 2A,B). In general, the narrowest VTA distance tends to be at the level of STJ. A VTC < 4 mm suggests a higher risk for coronary obstruction. A VTA < 2 mm suggests a high risk of sinus sequestration. Both situations are life-threatening and compromise blood flow to the coronaries. For short frame valves, it is sufficient to measure the inflow, outflow, and waist. For tall frame valves such as Evolut and NAVITOR, in addition to inflow and outflow measurements, the stent of the index prosthesis should be measured at several nodes. Nodes are visual points representative of half the length of each diamond-shaped cell on the tall frame valves. They are measured from the bottom of the valve (inflow) once the parallax is removed. Redo-TAVR valve sizing should be performed by measuring the in vivo area and perimeter at multiple levels within the frame of the valve. For example, for an Evolut valve, CTA measurements should be performed at nodes 1 to 6, understanding that the leaflets expand from node 3 to 6 (Figure 3). Depending on the implantation node, the area used for sizing is an average of that node and 3 mm below. For NAVITOR, valve measurements should be performed at nodes 1 and 2, as well as at the level of the commissural tabs (leaflets extend from node 1 to the commissural tabs). With the aforementioned information, one can choose the second prosthesis type (tall or short) and size, as well as an implantation depth to avoid coronary compromise.

It is common to describe a short-in-short or a tall-in-tall prosthesis combination with outflow to outflow or inflow to inflow. However, for a short-in-tall combination, the general nomenclature describes the outflow of the expanded second THV in comparison to the nodes of the index prosthesis. It is essential to note that the depth of prosthesis deployment may not be precise, and the final deployment can be either deeper or higher. The possibility of a stent frame under expansion of the index THV should always be considered. Finally, fluoroscopic angles can be provided to guide the removal of parallax and potential cusp identification, if needed, for leaflet modification.

### 4.1. Common CTA Terminology

Although there is no standardization of CTA terminology for redo-TAVR planning, some of the common terms described in the literature and relevant to procedure planning include:

### 4.2. Neoskirt

Neoskirt is defined as the portion of the 2nd THV that covers the deflected leaflets of the index THV, essentially creating a “tube graft”. The height of the neoskirt in relation to the native AV annulus and coronary ostia varies depending on the depth of implantation of both the index and redo-THV and the type of valve combination (short-in-short, short-in-long, long-in-short, and long-in-long). The height of the neoskirt will influence the risk of coronary obstruction during redo-TAVR and future coronary access.

### 4.3. Neoskirt Plane (NSP)

The superior edge of the neoskirt forms the neoskirt plane. Similar to the neoskirt, the level of NSP varies with valve combination and implantation depths, especially for a short-in-tall redo-TAVR procedure (Figure 2C,D). For the other three TAV-in-TAV combinations, there is much less variation in the implantation depth and hence NSP.

### 4.4. Coronary Risk Plane (CRP)

CRP is defined as the plane at the lower edge of the coronary ostia in relation to the index THV (Figure 2C,D). CRP varies depending on the native anatomy and the index prosthesis implant depth. When the NSP is below the CRP, the risk of coronary obstruction is low. However, when NSP is above the CRP, the risk of obstruction is higher. The CTA analysis should provide an assessment of this risk.

In patients with a narrow and low sinotubular junction (STJ) and thereby a narrow VTA distance, a high implantation of the 2nd THV may increase the risk of sinus sequestration. Furthermore, a BEV in an SEV (short-in-tall combination) may increase the risk of coronary obstruction or sequestration, as the radial force of BEV tends to expand the index SEV, further reducing the VTA/VTC distance and compromising coronary flow. In these patients, consideration should be given to tall-in-tall combination redo-TAVR and/or leaflet modification techniques.

With the constant need to decrease the incidence of pacemaker implantation after the index TAVR procedure, especially in younger patients, there has been a push for higher implantation of the THV. However, this renders a more complex redo-TAVR as the NSP will be higher and more likely above the CRP.

### 4.5. Leaflet Overhang

**Figure 4:**
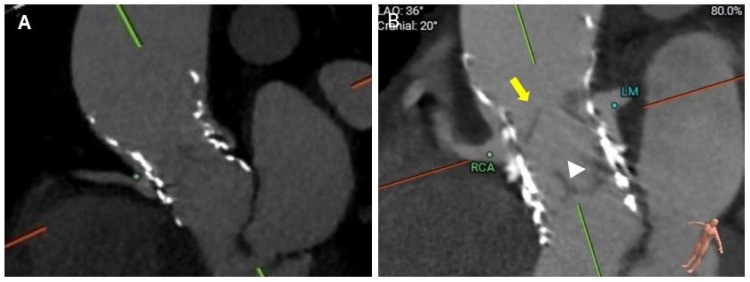
(**A**) Short-in-tall combination (S3 in Evolut) redo-TAVR in a patient with severe bioprosthetic AS. S3 implanted at node 6, demonstrating no leaflet overhang. (**B**) Short-in-tall combination (S3 in Evolut) redo-TAVR in a patient with severe bioprosthetic AR. S3 was implanted between nodes 3 and 4. Yellow arrow indicated a significant leaflet overhang. White arrowhead shows well-functioning S3 leaflets. AR, aortic regurgitation; AS, aortic stenosis; LM, Left main; RCA, Right coronary artery; TAVR, transcatheter aortic valve replacement.

Leaflet overhang refers to the portion of the index THV leaflets that extend above the 2nd THV after redo-TAVR (Figure 4, Video S1). Leaflet overhang is clinically relevant in patients undergoing short-in-tall redo-TAVR, especially with a Sapien S3 in Evolut. In other combinations of TAV-in-TAV (short-in-short, tall-in-tall, and tall-in-short), the 2nd TAVR valve will generally completely deflect the index valve leaflets. Deeper implantation of S3 in Evolut will result in greater leaflet overhang [23]. Excessive leaflet overhang may result in forward flow interference, especially when redo-TAVR is being considered for severe bioprosthetic AS with calcified and thickened leaflets. In patients with AR as the mechanism of index TAVR failure, this is less of a concern, although leaflet overhang may alter the blood flow in the sinuses, potentially creating slow-flow areas favorable for the development of HALT. In all cases, leaflet overhang may affect the hemodynamic performance of the valve and also its durability, and every attempt should be made to minimize the extent of leaflet overhang. Leaflet overhang may also impact future coronary access.

## 5. Procedural Considerations for Redo-TAVR

**Figure 5:**
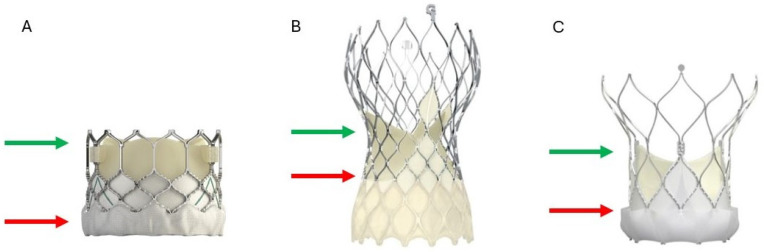
The nadir (red arrow) and top (green arrow) of the deflected leaflets in various valve flatforms. (**A**) For Sapien S3, the nadir of the leaflets is located 2–4 mm above the inflow, and the top of the deflected leaflets is located at the top of the commissure tab. (**B**) For the Evolut family valves, the nadir is located at node 3 and the top of the leaflets at node 6 (node 5 for the 23 mm valve). (**C**) For the NAVITOR valve, the nadir is at node 1, and the top of the leaflets is at the bottom of the commissure tab.

**Figure 6:**
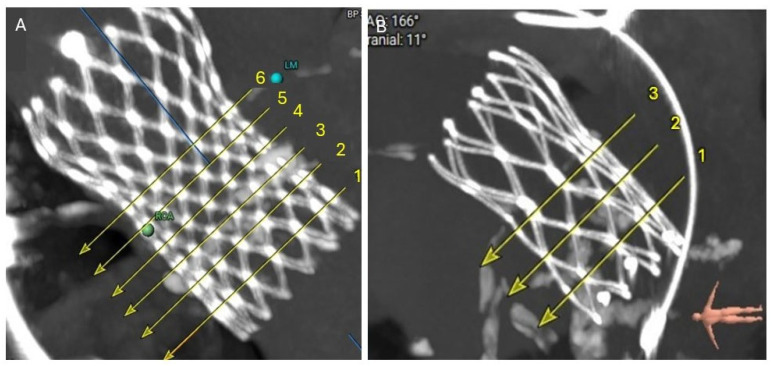
For tall THV frames, fluoroscopic markers (nodes) are commonly used for guiding the positioning of the second THV. For the Evolut valve (**A**), they are labeled as 1–9 (nodes 3–6 are commonly used for valve positioning). For the NAVITOR valve (**B**), they are labeled 1–3. THV, transcatheter heart valve.

The choice of the 2nd THV is based on the size, type, and implantation depth of the index THV, mode of bioprosthetic valve failure, commissural alignment, and the above-discussed CTA parameters. Fluoroscopic landmarks of the index THV may help guide the positioning of the 2nd THV. For short frame valves, fluoroscopic landmarks are simply the inflow and the outflow. For the S3 valve, the nadir of the leaflets is about 2–4 mm above the inflow, and the top of the deflected leaflets is at the top of the commissural tab (at the level of the stent frame for Sapien XT) (Figure 5A). However, for tall frames (both intra-annular and supra-annular), the nadir and top of the leaflets should be clearly defined. Both the Evolut and NAVITOR frames have fluoroscopic landmarks called nodes (Figure 6). For the Evolut valve, the nadir of the leaflets is at node 3, and the top of the deflected leaflets is at node 6 (node 5 for the 23 mm valve) (Figure 5B). For the NAVITOR valve, the nadir of the leaflets is at node 1, and the top of the deflected leaflets is at the bottom of the commissural tab (Figure 5C).

**Figure 7:**
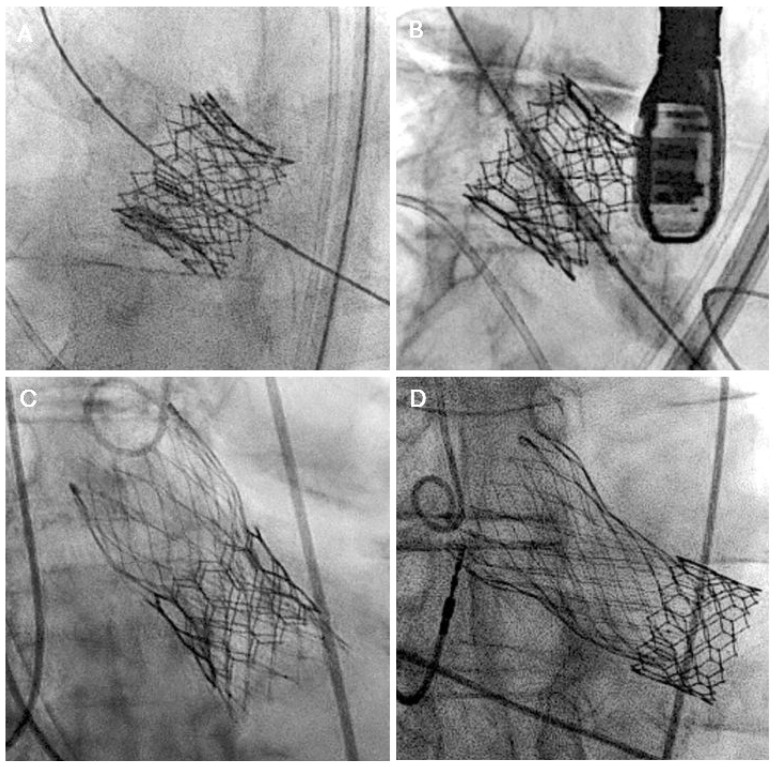
Short-in-short combination redo-TAVR: Sapien S3 in XT (**A**) and Sapien S3 in S3 (**B**) in patients with severe bioprosthetic AS. Long-in-short combination redo-TAVR: Evolut in Sapien S3 in a patient with severe bioprosthetic AS (**C**) and Evolut in S3 in a patient with late embolization of Sapien S3 (**D**). AS, aortic stenosis; TAVR, transcatheter aortic valve replacement.

When short-in-short redo-TAVR is planned, a valve size similar to the index THV, positioned inflow to inflow, is generally considered (Figure 7A,B). This also applies to tall-in-tall combination redo-TAVR. A slightly lower implantation with an acceptable degree of leaflet overhang is reasonable if the risk of coronary occlusion is high. Neoskirt is highest for the tall-in-tall combination, with a higher risk of coronary occlusion compared to other combinations. Therefore, further careful CTA assessment should be performed to measure VTA, CRP, and NSP to determine the need for coronary protection or leaflet modification techniques. Also, for a tall-in-tall combination, an optimal cell alignment is required to ensure future coronary access.

For a tall-in-short combination, the inflow of the 2nd THV is positioned at the inflow of the index THV (Figure 7C,D). Care should be taken not to position the valve lower in relation to the index THV, as it can constrain the THV, resulting in suboptimal leaflet expansion and possibly higher residual gradients. Valve sizing for this combination should be directed by CTA-assessed perimeter, as there can be nonuniform expansion of the index THV due to sequestration of native calcified and thickened leaflets.

**Figure 8:**
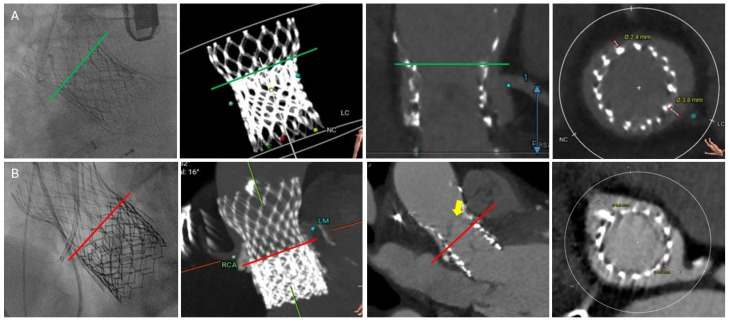
Short-in-tall combination redo-TAVR. (**A**): Sapien S3 implanted at node 7 (green line) of Evolut in a patient with bioprosthetic AS. VTA distance is adequate for both LM and RCA, maintaining coronary blood flow. (**B**): Sapien S3 in Evolut in a patient with severe AR. First S3 was landed at node 2, leading to moderate to severe residual AR. A second S3 was implanted between nodes 3 and 4 (red line). Significant leaflet overhang (yellow arrow) is seen post-redo-TAVR. AR, aortic regurgitation; AS, aortic stenosis; LM, left main; RCA, right coronary artery; TAVR, transcatheter aortic valve replacement; VTA, valve to aorta.

Lastly, for a short-in-tall combination of redo-TAVR, several factors need to be considered during the positioning of the 2nd THV. These include the mode of index THV failure, index valve size, coronary ostia heights, and VTA distance. BEV within a tall frame may expand the index THV stent frame, causing further sequestration of the sinuses, increasing the risk of coronary obstruction. For the Evolut index THV, the ideal implantation position of the 2nd THV is between nodes 4 and 6. A higher implantation is recommended if AS is the mode of valve failure to minimize leaflet overhang (Figure 8A). However, higher implantation may increase the risk of coronary occlusion. Pre-TAVR CTA simulations can help predict the risk of coronary occlusion at various implantation levels (node 3 to 6) and plan for mitigation strategies. On the contrary, a lower implantation can be considered if AR is the mode of failure prior to redo-TAVR, as some degree of leaflet overhang is acceptable in these patients (Figure 8B).

### 5.1. Coronary Occlusion Risk During Redo-TAVR and the Need for Protection

Coronary occlusion during redo-TAVR is rare, with a reported incidence of <1% [24,25]. This is likely due to careful patient selection and pre-procedure planning. CTA plays a crucial role in predicting coronary occlusion risk during redo-TAVR. The relationship between CRP and NSP helps determine the risk of coronary occlusion. If the CRP is above the NSP, the risk of coronary occlusion is minimal. When the CRP is below NSP, further assessment is needed to determine coronary occlusion risk. If the narrowest VTA distance is <2 mm (size of a 6FR coronary guide catheter), the risk is higher. This is more relevant in patients with long supra-annular index THV who undergo redo-TAVR with a BEV. Overlapping stent frames can reduce the effective cell orifice size, making coronary access by guide catheters technically challenging.

Several coronary occlusion mitigation strategies have been described in patients undergoing redo-TAVR. However, there is limited evidence supporting the use of these strategies in patients undergoing redo-TAVR. Some of these techniques include chimney stenting, BASILICA (Bioprosthetic Aortic Scallop Intentional Laceration to prevent Iatrogenic Coronary obstruction), UNICORN (undermining iatrogenic coronary obstruction with a radiofrequency needle), and lastly, the SURPLUS TAVR. Chimney stenting is particularly challenging and not the preferred strategy in redo-TAVR compared to TAVR-in-SAVR, as it entails placing a stent from the aorta into the left main artery or proximal right coronary, in between the stent frames of the THVs. This raises concerns for stent deformation and long-term patency. The BASILICA procedure, although originally described in TAVR-in-SAVR, is feasible in redo-TAVR. However, supportive evidence is scarce. Damlin et al. reported the first case of BASILICA in redo-TAVR in a patient with a supra-annular index THV [26]. Commissural alignment is a prerequisite for a successful BASILICA. In patients with commissural misalignment, BASILICA is challenging and may not provide any clinical benefit. On bench testing, Khan et al. reported less effective splaying of leaflets with BASILICA in newer generation THVs [27]. Also, if the commissures of the 2nd THV align unfavorably, BASILICA may be less effective in preventing coronary occlusion. The ShortCut (Pi-Cardia) is a dedicated leaflet splitting device used for valve-in-valve procedures. In the first-in-human report by Dvir et al., ShortCut was used for leaflet splitting in 2 patients with prior TAVR without evidence of coronary obstruction [28]. UNICORN and LLAMACORN (Leaflet Laceration with balloon Mediated Annihilation to prevent Coronary Obstruction with Radiofrequency Needle) are technically feasible in TAVR valves, but evidence is limited [29].

Coronary access after redo-TAVR can be particularly challenging, especially if commissural and coronary misalignment occurred during the index TAVR. A tall supra-annular THV frame, low sinus heights, and higher implantation depth are some other factors associated with challenging coronary access. Tang et al. analyzed Sapien S3 in Evolut (short-in-tall) combination redo-TAVR at two different positions (nodes 4 and 5) and demonstrated that coronary accessibility improved when the outflow of S3 was positioned at node 4 with an initial Evolut implant depth of 5 mm [30]. Moreover, TAVR is associated with a small but significant reduction in coronary fractional flow reserve and resting flow ratios immediately post-procedure [31]. In patients with severe CAD who are candidates for TAVR, there may be a possible benefit of PCI before TAVR. The NOTION-3 trial showed that among patients with CAD undergoing TAVR, PCI was associated with a lower risk of composite all-cause death, MI, or urgent revascularization at 2 years compared to conservative management [32]. In younger patients with severe CAD undergoing TAVR, a PCI-first, followed by TAVR strategy may minimize coronary inaccessibility in the future.

### 5.2. Patient Prosthesis Mismatch (PPM)

PPM is a significant finding after redo-TAVR due to the increasingly smaller inflow diameter of the 2nd THV inside a previously existing index THV. In the VIVID registry (TAVR-in-SAVR), a significant proportion of patients developed moderate or more PPM post-TAVR [33]. However, the incidence of moderate (5.8%) and severe (2.9%) PPM after redo-TAVR was relatively lower as reported in the international TRANSIT registry [25]. Within this cohort of patients, the incidence was higher in patients who underwent redo-TAVR for a stenotic index THV compared to a severely regurgitant valve. Patients with a larger body surface area who undergo redo-TAVR with a small BEV are particularly susceptible to developing PPM. A supra-annular SEV is a reasonable THV of choice in these patients to minimize the incidence and severity of PPM.

### 5.3. Stroke Risk During Redo-TAVR

Stroke after TAVR is associated with substantial morbidity and mortality, with studies demonstrating a 3-fold increase in 30-day mortality [34]. Despite improvements in procedural techniques, overall stroke rates remained fairly constant with only modest reductions. Results from the transcatheter valve technology (TVT) registry showed that between 2011 and 2019, in-hospital and 30-day stroke rates reduced from 2.1% to 1.6% and 2.75% to 2.3%, respectively [35]. While there is a lack of prospective data, retrospective observational studies evaluating outcomes of redo-TAVR have reported similar 30-day stroke rates with an overall incidence ranging between 1.4 and 3.5% [24,25,36,37]. There is currently a lack of evidence supporting the routine use of cerebral embolic protection devices in patients undergoing TAVR [38,39]. Whether cerebral embolic protection is associated with favorable outcomes in redo-TAVR is unknown.

### 5.4. Pacemaker After Redo-TAVR

Permanent pacemaker placement, although trending downward over time due to refinements in pre-procedural assessments and valve implantation techniques, is still a major complication of TAVR. Incidence of permanent pacemaker placement after TAVR stands at ~10% [35]. In the VIVID registry, the rate of permanent pacemaker implantation after TAVR-in-SAVR was lower at 6.4%, in part due to the protection offered by the surgical valve frame [33]. A similar trend has been reported in patients undergoing redo-TAVR. Observational and registry studies demonstrated a pacemaker rate of 4 to 11%.

## 6. Post-Procedural Considerations

### Antithrombotic Therapy

Antiplatelet therapy (single vs. dual) after TAVR has been a topic of debate. In valve-in-valve, the presence of extra overlapping stents (typically slightly smaller than the index THV) and higher residual gradients (compared to index TAVR) may predispose the valve to thrombosis and structural deterioration. However, evidence for this after redo-TAVR is currently lacking due to a paucity of long-term studies. The incidence of HALT after redo-TAVR is unknown [40]. Routine CTA at 3–6 months follow-up may provide more insights into the true incidence of HALT after redo-TAVR and may help guide antithrombotic therapy. Until further data are available, prophylactic antithrombotic therapy post-redo-TAVR should be individualized.

## 7. Clinical Evidence for Redo-TAVR

There is a scarcity of prospective data supporting redo-TAVR for patients with TAVR valve degeneration. Most evidence on redo-TAVR is limited to observational studies and registry data (Table 1).

Schmidt et al. first reported outcomes of redo-TAVR in 19 patients at two German centers who underwent index TAVR between 2008 and 2015 [41]. AR was the most common mode of index THV failure (16/19 patients). Of the 19 patients, 16 had index TAVR with a SEV and 3 with a BEV. Redo-TAVR success rate was 89% with minimal post-procedural gradients and no PPM. However, one-year all-cause mortality was high at 33% due to the high-risk nature of the population.

In a multi-center retrospective study of 13,876 TAVR patients, Barbanti et al. reported a redo-TAVR rate of 0.4% [36]. Indications for redo-TAVR were BVD (AS and AR) in 50% of patients and paravalvular leak (PVL) in the rest. SEV was the most common THV at index TAVR (76%). The mean interval from index TAVR to redo-TAVR was 812 ± 750 days. In 80% of the patients, redo-TAVR was performed with the identical valve type (SEV in SEV and BEV in BEV). Redo-TAVR was successful in reducing PVL to mild or less in 92% of patients. Survival was 85.1% at 635 days after redo-TAVR.

In a large registry from 37 centers, including 63,876 patients who underwent TAVR, Landes et al. reported a very low rate of redo-TAVR at 0.33% [24]. Of these, 64% underwent redo-TAVR one year or later after the index TAVR procedure (termed probable THV failure due to PVL, valve thrombosis, or BVD), while the rest of the 36% underwent redo-TAVR within one year (termed probable TAVR procedural failure due to device malpositioning, embolization). Of the patients with probable THV failure, the mode of presentation was pure AS in 37%, and pure AR in 30%, while the rest constituted mixed etiology. The median time from index TAVR to redo-TAVR was 5 years, which is significantly shorter than the median 9-year interval seen after SAVR in the VIVID registry [42]. In contrast, those with probable procedural failure presented mostly with AR. In the overall cohort, 61% of patients underwent index TAVR with SEVs. At redo-TAVR, 50% of patients were treated with SEVs. Sapien S3 was the most frequently used THV at redo-TAVR. Similar types of THVs were used in 59% of patients (SEV in SEV [35%] and BEV in BEV [24%]), while SEV in BEV was used in 15% and BEV in SEV was used in 26% of patients. The overall procedural success rate of redo-TAVR was 85.1%, mostly attributed to the high residual gradients seen in 14% of the patients and ≥moderate residual AR seen in 9% of patients. Interestingly, coronary obstruction was seen in just 0.9% of the cases, which likely reflects careful pre- and intra-procedural planning in this complex subset of patients. Survival at 30 days and 1 year was 97.2% and 86.5%, respectively.

Among 133,250 Medicare beneficiaries who underwent TAVR between 2012 and 2017, Percy et al. reported a subsequent repeat TAVR rate of 0.46% [37]. In this study, the median time from index TAVR to redo-TAVR was only 154 days (IQR: 58–537 days). While the study does not specify the reasons for redo-TAVR, due to a lack of comprehensive imaging data, the short interval between index and repeat procedures suggests possible THV procedural failure in a majority of the patients. All-cause mortality was 6% and 22% at 30 days and one year, respectively. The study further compared redo-TAVR with TAVR explantation using propensity matching. Redo-TAVR was associated with lower 30-day mortality compared to TAVR explantation (6.2% vs. 12.3%), albeit with similar one-year mortality rates (21% vs. 20.8%). The incidence of MACE at 30 days is significantly higher with TAVR explantation. As the study included patients who underwent index TAVR between 2012 and 2017, the study findings are not pertinent to patients enrolled in the low-risk TAVR trials.

Lastly, the international TRANSIT registry included 172 patients with degenerative THVs who underwent a second TAVR procedure. The indication for a second TAVR was AS in 33%, AR in 56%, and mixed etiology in 11% of patients [25]. Redo-TAVR success rate was 79% mainly due to high residual gradients (14%) and AR (7%). The choice of the second TAVR valve was a SEV in 61% of patients. In-hospital mortality was high at 4.1%, all attributed to cardiovascular causes, but thirty-day all-cause mortality among patients alive at discharge was acceptable at 2.9%. There were no cases of coronary obstruction in this study, indicating more careful patient selection. These studies established redo-TAVR as a relatively safe and effective strategy in select patients with index THV dysfunction. Prospective studies comparing redo-TAVR with SAVR + THV explantation are much needed to better define the ideal treatment strategy for low-risk patients with THV dysfunction.

### Future Perspectives

Most evidence on redo-TAVR is currently limited to retrospective studies and registries, which highlights the need for prospective studies with well-defined enrollment criteria and longitudinal follow-up. The REdo tranScatheter Aortic Valve Replacement for Transcatheter aOrtic Valve failuRE (RESTORE) trial (NCT06777368) is a prospective multi-center study designed to evaluate the outcomes of redo-TAVR in patients with BVD. In this study, patients who underwent index TAVR with an Evolut SEV or SAPIEN BEV with evidence of BVD are included. Valves of choice for redo-TAVR include either an Evolut or an S3, at the discretion of the implanting physician. Primary outcomes include 30-day redo-TAVR success, freedom from all-cause mortality, coronary obstruction, unplanned coronary revascularization, device-related surgery, or repeat intervention, as well as the composite endpoint of 1-year freedom from all-cause mortality, any stroke, and any valve-related rehospitalization. The trial is currently enrolling patients.

The ReTAVI registry (NCT05601453) is a multicenter, prospective observational registry currently enrolling patients at 62 centers across Europe. Patients aged 18 years and older with evidence of index THV device failure, regardless of SVD severity, will be enrolled in this registry. The valve of choice for redo-TAVR is Sapien S3/S3 ULTRA. The study plans to enroll approximately 150 patients. Primary outcomes include 30-day device success, which includes technical success, freedom from mortality, freedom from surgery or intervention related to the device, and a mean gradient < 20 mm Hg. The durability of the 2nd THV will be assessed at 12 months. These registry data may help in understanding the clinical and procedural characteristics associated with technical success and outcomes of redo-TAVR.

With the latest American [43] and European [44] guidelines lowering the recommended age threshold for TAVR to 65 years and 70 years, respectively, and expanded indications, TAVR adoption is expected to increase. Consideration should be given to the “lifetime management” of AS and durability of THV, as a significant proportion of these patients may eventually require repeat transcatheter procedures for BVD. This also lays extreme emphasis on “getting it right the first time”. Careful pre-procedural and procedural planning is crucial to the success of index TAVR. Commissural and coronary alignment during index THV is crucial for future coronary access in the event of an acute coronary syndrome. Tall supra-annular THVs carry a higher risk of difficult or nonselective coronary engagement compared to a short frame THV, especially if they are commissurally misaligned. All these variables influence the choice of THV during the index TAVR procedure. The implantation technique also matters. A lower implantation depth of THV, in particular for tall-frame valves, is usually preferred for redo-TAVR, as this will subsequently lower the neoskirt plane and reduce the risk of coronary obstruction during redo-TAVR. However, a lower index THV implantation depth should be balanced with the increased risk of PPM.

## 8. Conclusions

With TAVR being increasingly performed in low to intermediate-risk patients, redo-TAVR is expected to see an upward trend in the coming years. Understanding the native AV anatomy and the index THV characteristics is vital for the procedural success of redo-TAVR. Pre-procedure CTA plays a significant role in identifying patients at risk for coronary obstruction and also aids in determining the ideal valve combination for optimal patient outcomes.

## Figures and Tables

**Table 1:** Summary of evidence on redo-TAVR.

Study (Year)	Number of Patients	Index THV	Mode of Failure	2nd THV	Time Between Index TAVR and Redo-TAVR	All-Cause Mortality	Permanent Pacemaker	Coronary Obstruction	Stroke (30 Days)
Schmidt et al. (2016) [41]	19	SEV = 84% BEV = 16%	AR = 84% AS = 16%	SEV = 37% BEV = 63%	644 (191–1831) days	33% at 1 yr	11%	None	5%
Barbanti et al. (2016) [36]	50	SEV = 76% BEV = 24%	AR = 76% AS = 18% Mixed = 6%	SEV = 60% BEV = 40%	812 ± 750 days	14.9% at 2 years	8.6%	2%	2%
Landes et al. (2020) [24]	212	SEV = 61.4% BEV = 38.6%	AR = 45% AS = 30% Mixed = 25%	SEV = 50% BEV = 50%	2 days to 11.6 yrs	2.9% at 30 days	9.6%	0.9%	1.4%
Percy et al. (2020) [37]	617	N/A	N/A	N/A	154 (58–537) days	22% at 1 yr	4.2%	N/A	1.8%
Testa et al. (2021) [25]	172	SEV = 65% BEV = 35%	AR = 56% AS = 33% Mixed = 11%	SEV = 61% BEV = 39%	268–1323 days (reported by valve type)	10% at 1 yr	4.1%	0%	3.5%

## Data Availability

Not applicable. This is a narrative review.

## Supplementary Materials

The following supporting information can be downloaded at: https://www.mdpi.com/article/10.3390/jcm14186608/s1, Video S1: Cardiac CTA cine showing significant leaflet overhang after redo-TAVR with Sapien S3 landed between node 2 and 3 in a patient with a failed Evolut with severe AR. AR, aortic regurgitation; CTA, computed tomography angiography; TAVR, transcatheter aortic valve replacement.

## Abbreviations

AS	Aortic stenosis
AR	Aortic regurgitation
BEV	balloon expandable valve
BVD	Bioprosthetic valve dysfunction
CRP	Coronary risk plane
CTA	Computed tomography angiography
HALT	Hypoattenuated leaflet thickening
NSP	Neoskirt plane
PPM	Patient prosthesis mismatch
PVL	Paravalvular leak
SAVR	Surgical aortic valve replacement
SEV	Self-expanding valve
SVD	Structural valve deterioration
TAVR	Transcatheter aortic valve replacement
THV	Transcatheter heart valve
VTA	Valve to aorta
VTC	Valve to coronary
VTJ	Valve to sinotubular junction
